# Effects of cigarette smoking on metabolic activity of lung cancer on baseline ^18^F-FDG PET/CT

**DOI:** 10.7717/peerj.13352

**Published:** 2022-04-25

**Authors:** Maoqing Jiang, Xiuyu Guo, Xiaohui Zhang, Qiaoling Gao, Weiqi Mei, Jingfeng Zhang, Jianjun Zheng

**Affiliations:** 1 Department of PET/CT center, Hwa Mei Hospital, University of Chinese Academy of Sciences, Ningbo, Zhejiang, China; 2 Department of Nuclear Medicine, Hwa Mei Hospital, University of Chinese Academy of Sciences, Ningbo, Zhejiang, China; 3 Department of Education, Hwa Mei Hospital, University of Chinese Academy of Sciences, Ningbo, Zhejiang, China

**Keywords:** Cigarette smoking, Metabolic status, PET/CT, 18F-FDG, Lung cancer

## Abstract

**Background:**

Never-smokers with lung cancer usually have a higher survival rate than that of smokers. The high metabolic activity of lung cancer on ^18^F-2-Fluoro-2-deoxyglucose (^18^F-FDG) PET/CT generally indicates a poor outcome. However, there is a lack of reports on the association between cigarette smoking and ^18^F-FDG metabolic activity in patients with lung cancer. In this study, we aimed to investigate the effects of cigarette smoking on metabolic activity of lung cancer on ^18^F-FDG PET/CT.

**Materials and Methods:**

A total of 338 patients (230 males, 108 females; mean age: 66.3, range 34–86) with pathologically diagnosed lung cancer were enrolled from September 2019 to April 2021. All patients underwent baseline ^18^F-FDG PET/CT and the maximum standard uptake value (SUVmax) of the primary tumor (pSUVmax), lymph node (nSUVmax) and distant metastasis (mSUVmax) were measured. The associations between cigarette smoking status, clinical stage, pathological subtypes and metabolic parameters on ^18^F-FDG PET/CT were analyzed.

**Results:**

Of the 338 patients, cigarette smoking was identified in 153 patients (45.3%) and the remaining 185 (54.7%) were never-smokers. Smoking was found more frequently in males, squamous cell carcinoma (SCC) and stage III–IV diseases. The pSUVmax in smokers was significantly higher than that in never-smokers (*t* = 3.386, *P* < 0.001), but the nSUVmax and mSUVmax revealed no statistically significant differences (*t* = 0.399, *P* = 0.690 and *t* = 0.057, *P* = 0.955; respectively). With the increase of cumulative smoking dose, pSUVmax increased significantly (*r* = 0.217, *P* < 0.001). In addition, the pSUVmax in patients with stage III–IV was significantly higher than that in stage I–II (*t* = 8.509, *P* < 0.001). Smokers showed a higher pSUVmax than never-smokers for patients with stage I–II (*t* = 3.106, *P* = 0.002), but not in stage III–IV (*t* = 0.493, *P* = 0.622). The pSUVmax was significantly different among patients with different pathological subtypes of lung cancer (*F* = 11.45, *P* < 0.001), while only the adenocarcinoma (ADC) and SCC groups showed a difference in pSUVmax (*t* = 6.667, *P* < 0.001). Smokers with ADC showed a higher pSUVmax when compared to never-smokers, but not in SCC. There were no significant differences of pSUVmax between smokers and never-smokers at stage I–II ADC or SCC and stage III–IV ADC or SCC.

**Conclusions:**

This study demonstrated a close association between cigarette smoking and the metabolic activity of lung cancer and suggests that smoking may be a potential risk factor of higher pSUVmax in early lung cancer on ^18^F-FDG PET/CT.

## Introduction

Despite improvements in diagnosis and treatment modalities, lung cancer remains one of the leading causes of cancer-related deaths worldwide (Siegel, Miller & Jemal, 2020). It was estimated that there were approximately 228,820 newly-diagnosed lung cancer cases and 135,720 deaths from lung cancer in the United States in 2020 (Siegel, Miller & Jemal, 2020). Obviously, cigarette smoking is one of the major risk factors for the development of lung cancer and accounts for about 85%–90% of lung-cancer related deaths in the USA (Centers for Disease Control and Prevention, 2005). However, around 25% of lung cancer cases worldwide were not correlated with tobacco smoking (Parkin et al., 2005). The molecular genetics, clinicopathological features, and survival rates of patients with lung cancer between smokers and never-smokers were significantly different (Yano et al., 2008; Nordquist et al., 2004). Thus, lung cancers in smokers and never-smokers are proposed to be separate entities (Sun, Schiller & Gazdar, 2007; Toh et al., 2006).

Epidermal growth factor receptor (EGFR), a receptor tyrosine kinase, has been demonstrated to be highly expressed in many epithelial tumors, including lung cancer (Ichihara et al., 2007). EGFR-tyrosine kinase inhibitors (TKIs) therapy is the mainstay treatment modality for patients with locally-advanced unresectable or metastatic non-small cell lung cancer (NSCLC) (Park et al., 2020; Wu et al., 2019). Patients with EGFR mutations tend to have higher response rates and longer progression free survival (PFS) when treated with TKIs, as compared to those treated with conventional cytotoxic chemotherapy (Maemondo et al., 2010; Sequist et al., 2013). Interestingly, EGFR mutations in patients with NSCLC were significantly higher in never-smokers than in smokers (Shigematsu & Gazdar, 2006). The overall survival (OS) of never-smoking NSCLC patients was superior to that of smokers (Yano et al., 2008).

Positron emission tomography/computed tomography (PET/CT) with ^18^F-2-Fluoro-2-deoxyglucose (^18^F-FDG), a noninvasive and functional imaging method, has been widely used for evaluating outcome for patients with NSCLC and has a powerful ability to predict the mutation status of EGFR in NSCLC (Berghmans et al., 2008; Zhang et al., 2020). The maximal standard uptake value (SUVmax) is a promising semiquantitative parameter to reflect the metabolic activity of NSCLC on ^18^F-FDG PET/CT (Moon et al., 2013). It has been reported that high SUVmax of primary tumor on ^18^F-FDG PET/CT is associated with shorter PFS and/or OS in patients with NSCLC (Nappi et al., 2015; Roengvoraphoj et al., 2020). Patients with NSCLC harboring EGFR mutations often showed lower SUVmax than those with wild-type EGFR (Lv et al., 2018).

In brief, never-smoking NSCLC patients often present with high EGFR mutation status, and patients with NSCLC harboring EGFR mutations usually show low SUVmax of the primary tumor. However, there is a lack of reports on the association between cigarette smoking history and ^18^F-FDG metabolic activity on PET/CT in patients with lung cancer. Thus, in this study, we aimed to investigate the correlation between cigarette smoking status and the ^18^F-FDG metabolic activity of lung cancer.

## Materials & Methods

### Patients and data collection

**Figure 1:**
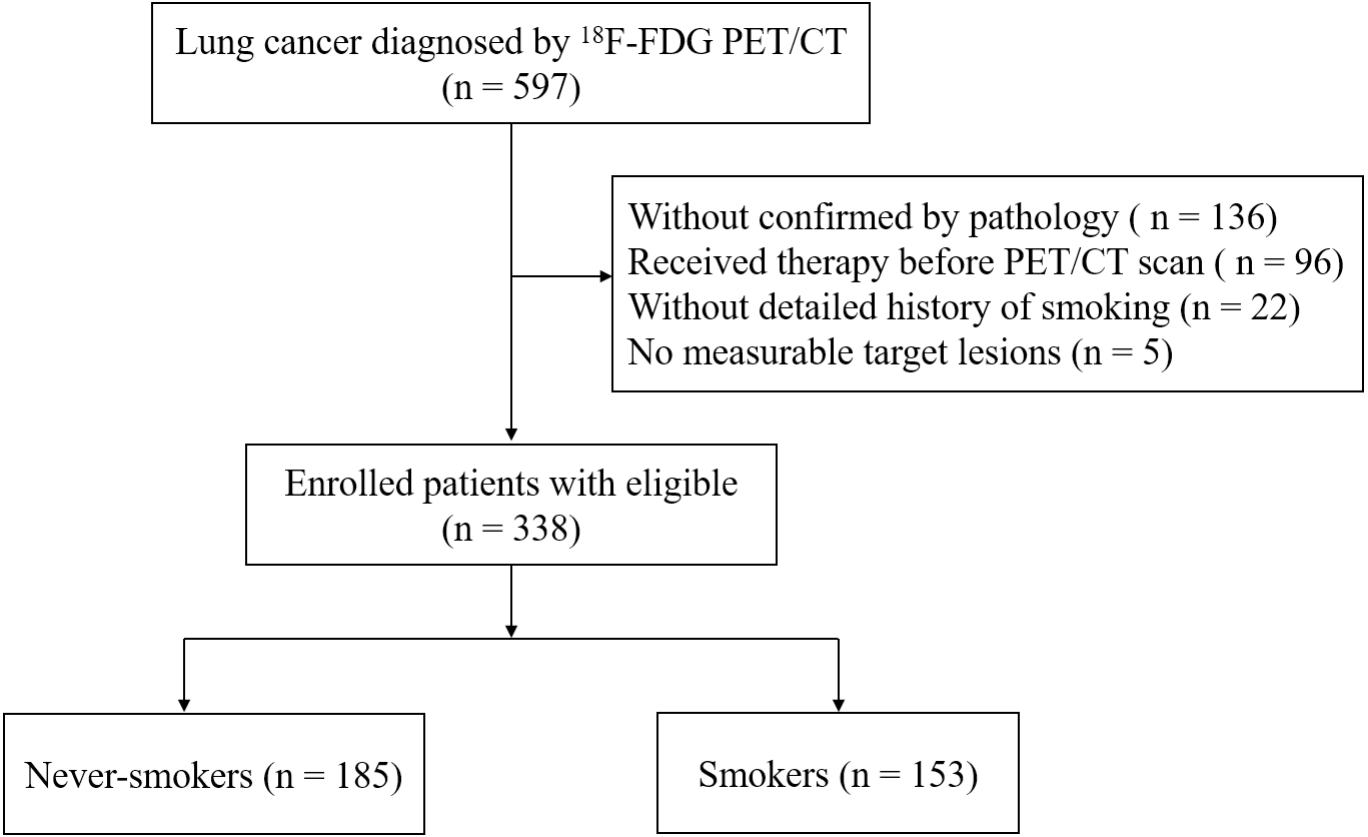
Flowchart of patient selection.

From September 2019 to April 2021, patients with primary lung cancer diagnosed histopathologically at Hwa Mei Hospital, University of Chinese Academy of Sciences (Ningbo, China) were analyzed. Initially, we evaluated 597 patients with lung cancer that diagnosed by ^18^F-FDG PET/CT imaging. Inclusion criteria included: (i) ≥ one measurable target lesion on CT scan; (ii) underwent ^18^F-FDG PET/CT before initiation of any treatment, *e.g.*, conventional cytotoxic chemotherapy, adjuvant chemoradiotherapy, targeted EGFR mutation therapy, or surgery; (iii) histopathology confirmed lung cancer, including adenocarcinoma (ADC), squamous cell carcinoma (SCC), large cell carcinoma, small cell carcinoma and others; (iv) a detailed history of cigarette smoking. In the end, a total of 338 patients met the requirements and were enrolled in our study (Fig. 1). The clinicopathological features, including age, gender, cigarette smoking status, the histopathological subtype of lung cancer and clinical stage were summarized in Table 1.

**Table 1:** Patient characteristics.

Variables	Total patients	Never-smokers		Smokers		*P* value
		No.	%	No.	%	
Sex (*n* = 338)						
Male	230	85	45.9	145	94.8	<0.001
Female	108	100	54.1	8	5.2	
Age at diagnosis, years						
No. of patients	338	185	54.7	153	45.3	
Median ± SD	66.3 ± 8.8	65.6 ± 9.7		67.2 ± 7.6		0.104
Range	34–86	34–85		50–86		
Histological subtype (*n* = 338)						
Adenocarcinoma	203	134	72.4	69	45.1	<0.001
Squamous cell carcinoma	85	30	16.2	55	35.9	
Large cell carcinoma	6	3	1.6	3	2.0	
Small cell carcinoma	31	11	5.9	20	13.1	
Others	13	7	3.9	6	3.9	
Clinical stage (*n* = 338)						
I	118	74	40.0	44	28.8	0.004
II	37	24	13.0	13	8.5	
III	77	30	16.2	47	30.7	
IV	106	57	30.8	49	32.0	
SUVmax on ^18^F-FDG PET/CT						
Primary tumor (*n* = 338)	11.2 ± 6.2	10.1 ± 6.2		12.4 ± 5.9		<0.001
Metastatic lymph node (*n* = 169)	10.1 ± 5.4	9.9 ± 5.8		10.3 ± 5.0		0.690
Distant metastasis (*n* = 106)	10.0 ± 6.3	10.0 ± 6.8		10.1 ± 5.8		0.955

The study has been approved by the Institutional Review Board (IRB) of Hwa Mei Hospital, University of Chinese Academy of Sciences, protocol number YJ-NBEY-KY202108401, and the need for written informed consent was waived due the retrospective nature of this study.

### Assessment of cigarette smoking status

The status of cigarette smoking was classified into two subgroups, never-smokers and smokers. Never-smokers were strictly defined as patients who smoked fewer than 100 cigarettes in their lifetime and smokers were defined as those who smoked more than 100 cigarettes, regardless of smoked before or current (Kawaguchi et al., 2010a; Kawaguchi et al., 2010b; Pham et al., 2006). The pack-year index, as a parameter to embody cumulative smoking dose, which is calculated by multiplying smoking period (years) and the number of cigarette packs smoked per day, is an important factor to reflect the risk of developing lung cancer (Alberg et al., 2007). One pack equals 20 cigarette equivalents, 1 pack-year equals 1 pack/day for 1 year, and so on.

### Technique of ^18^F-FDG whole-body PET/CT scan

All patients underwent ^18^F-FDG PET/CT scan and PET/CT images were obtained by using a GE Discovery 710 PET scanner (GE Healthcare, Milwaukee, Wisconsin, USA) after at least 6 h of fasting (Boellaard et al., 2010). The peripheral blood glucose level was detected to ensure that the values were within normal range (<7.0 mmol/L) before intravenous administration of ^18^F-FDG. Three-dimensional mode PET/CT scans from skull base to upper thigh were initiated 45 to 60 min after injection of 5.2–7.4 MBq/kg ^18^F-FDG. A low-dose spiral CT scan (140 kV, 10 mA, 0.5 s rotation time and 40 mm collimation) was conducted at the start of imaging to provide PET attenuation correction and anatomical reference. The attenuation-corrected PET image was scanned at 2.5 min per bed position and reconstructed using CT data with iterative algorithms. Image readouts were obtained on a Xeleris Workstation (GE Healthcare), which could display PET, CT and fusion PET/CT images in transverse, sagittal and coronal planes.

### ^18^F-FDG PET/CT analysis

All PET and CT images were evaluated in consensus by two experienced nuclear physicians who knew the clinical data. Abnormal uptake of ^18^F-FDG in lesions was defined as that their metabolic activity was greater than that of the surrounding background, and the intensity of ^18^F-FDG uptake was quantified by calculating the maximum standard uptake value (SUVmax). The region of interest (ROI) around the primary tumor with abnormal uptake of ^18^F-FDG was manually drawn on the transverse image. SUV was calculated from the attenuation-corrected regional images which based on the amount of injected ^18^F-FDG, total body weight, and soft-tissue uptake. SUV = (activity/unit volume)/(injected dose/total body weight). SUVmax was defined as the peak SUV with the highest counts on a pixel within ROI to quantify the uptake of ^18^F-FDG. According to visual qualitative analysis, metastatic lymph nodes were defined as lymph nodes with higher metabolic activity than the background mediastinal blood pool (Lee et al., 2015).

### Statistical analysis

Descriptive statistics were performed on the demographic data of patients. The quantitative data were expressed as mean ± standard deviation (SD). Continuous variables, such as the differences of the maximum standard uptake value of the primary tumor (pSUVmax), lymph node (nSUVmax) and distant metastasis (mSUVmax) between smokers and never-smokers, were analyzed by unpaired Student’s *t*-test. The frequency of gender (male *vs.* female), histopathological patterns (ADC *vs.* SCC) and clinical stage (I–II *vs.* III–IV) were compared between smokers and never-smokers using Fisher’s exact test analysis. The correlation between cumulative smoking dose and pSUVmax in smokers was analyzed by linear regression analysis. One-way analysis of variance and Bonferroni’s multiple comparison test were used to evaluate the associations between pSUVmax and different pathological subtypes of lung cancer. A two-sided *P*-value of <0.05 was considered statistically significant. All statistical analyses and graph designs were performed using GraphPad Prism 5.1 software (GraphPad Software, Inc., San Diego, CA, USA).

## Results

### Patients’ characteristics

The characteristics of 338 patients stratified by cigarette smoking history are listed in Table 1. Among the 338 patients, 185 (54.7%) were never-smokers and 153 (45.3%) were smokers. In terms of gender, the majority of smokers were male compared with those in never-smokers (94.8% *vs.* 45.9%, *P* < 0.001). No significant difference was observed in the mean age at diagnosis of lung cancer between never-smokers and smokers (65.6 ± 9.7 *vs.* 67.2 ± 7.6, *P* = 0.104).

Regarding histopathological types, never-smokers had a higher proportion of ADC compared with smokers (72.4% *vs.* 54.1%, *P* < 0.001), while smokers had a higher proportion of SCC (35.9% *vs.* 16.2%, *P* < 0.001). In addition, 155 presented with early stage (I–II) and the remaining 183 presented with advanced stage (III–IV). Compared with never-smokers, smokers had a higher proportion of advanced lung cancer at the time of diagnosis (62.7% *vs.* 47.0%, *P* = 0.004).

### Associations between cigarette smoking and ^18^F-FDG uptake in primary lung cancer, metastatic lymph nodes and distant metastases

**Figure 2:**
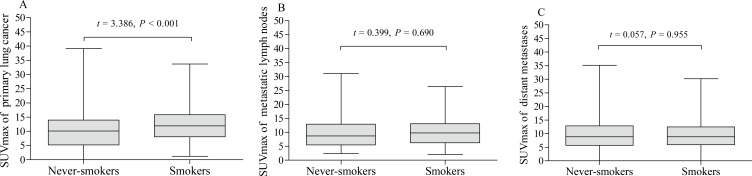
Metabolic activity of lung cancer on ^18^F-FDG PET/CT. The levels of pSUVmax (A), nSUVmax (B) and mSUVmax (C) on ^18^F-FDG PET/CT were compared according to cigarette smoking history of patients with lung cancer (never-smokers *vs.* smokers).

**Figure 3:**
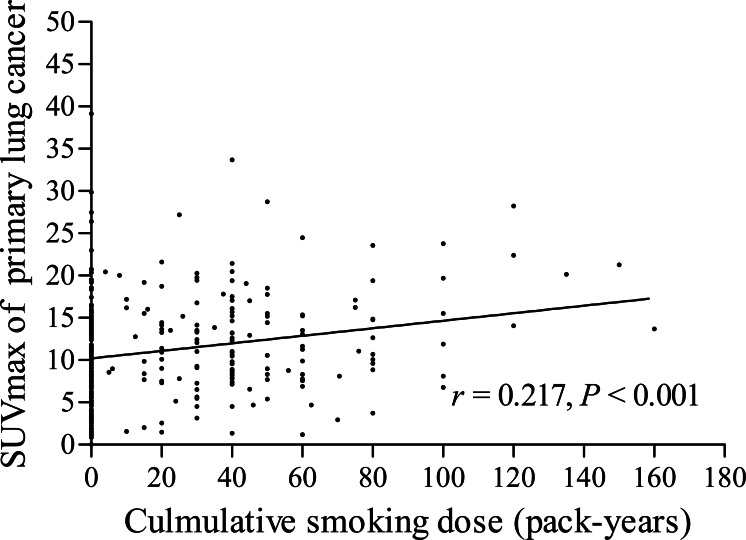
Correlation between cumulative smoking dose and pSUVmax. A significant correlation was observed between cumulative smoking dose (pack-years) and pSUVmax on ^18^F-FDG PET/CT in patients with lung cancer (*r* = 0.217, *P* < 0.001).

The pSUVmax was significant higher in smokers than that in never-smokers (12.4 ± 5.9 *vs.* 10.1 ± 6.2; *t* = 3.386, *P* < 0.001, Fig. 2A). We further analyzed the correlation between cumulative smoking dose and the metabolic activity of the primary lung cancer with linear regression analysis, which showed a significant correlation between cigarette pack-years and pSUVmax (*r* = 0.217, *P* < 0.001, Fig. 3). Moreover, the changes of pSUVmax in smokers by pack-years were also evaluated, but no significant difference of pSUVmax was observed between pack-years <40 and pack-years ≥ 40 (*t* = 0.608, *P* = 0.544).

Of the 338 patients, 169 (50%) had metastatic lymph nodes on ^18^F-FDG PET/CT scan, including 81 never-smokers and 88 smokers. Compared with smokers, never-smokers had a lower rate of lymph node metastasis (*P* = 0.016, Table 2), which was consistent with clinical stage, but there was no statistically significant difference in distant metastasis (*P* = 0.640, Table 2). In addition, the nSUVmax and mSUVmax revealed no significant differences between smokers and never-smokers (Table 1, Figs. 2B and 2C).

**Table 2:** Comparison of metastatic sites between never-smokers and smokers.

Locations of metastasis	No. of never-smokers	No. of smokers	*P* value
Patients with metastatic lymph nodes			
Yes	81	88	0.016
No	104	65	
Patients with distant metastasis			
Yes	56	50	0.640
No	129	103	

### Associations between pSUVmax, clinical stage, pathological subtypes and cigarette smoking

**Figure 4:**
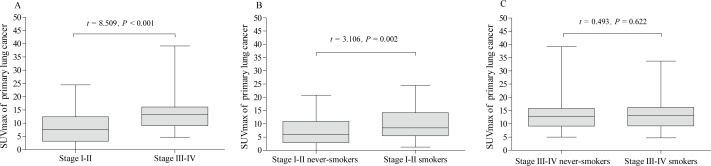
Comparative analysis of pSUVmax in clinical stage and cigarette smoking history. The pSUVmax was significantly higher in patients with stage III–IV than those in stage I–II (A). The pSUVmax in stage I–II smokers was significantly higher than that in never-smokers (B), but not in stage III–IV smokers (C).

The pSUVmax in clinical stage III–IV was significantly higher than that in stage I–II (13.5 ± 5.6 *vs.* 8.2 ±5.7; *t* = 8.509, *P* <0.001, Fig. 4A). In addition, the pSUVmax in smokers was higher than that in never-smokers for patients with stage I–II (10.1 ± 6.1 *vs.* 7.1 ± 5.1; *t* = 3.106, *P* = 0.002, Fig. 4B), but not in stage III–IV (13.7 ± 5.5 *vs.* 13.3 ± 5.7; *t* = 0.493, *P* = 0.622, Fig. 4C).

**Figure 5:**
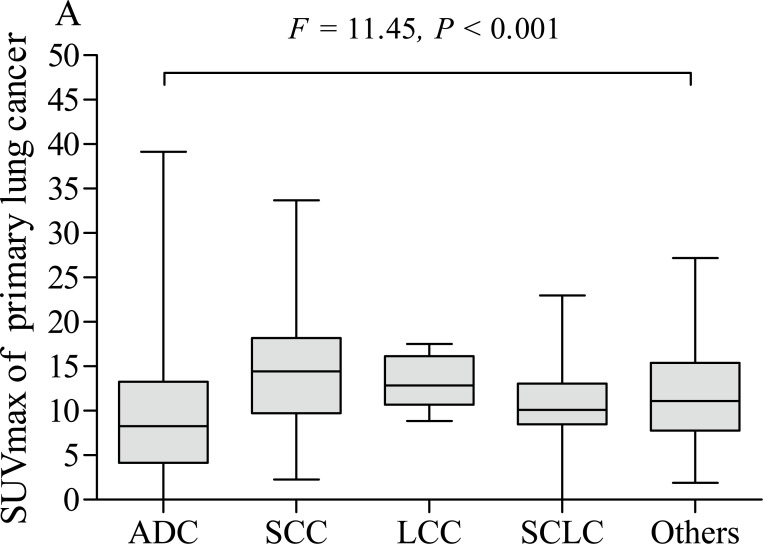
Comparative analysis of pSUVmax among different pathological subtypes. ADC, adenocarcinoma; SCC, squamous cell carcinoma; LCC, large cell carcinoma; SCLC, small cell lung cancer.

**Figure 6:**
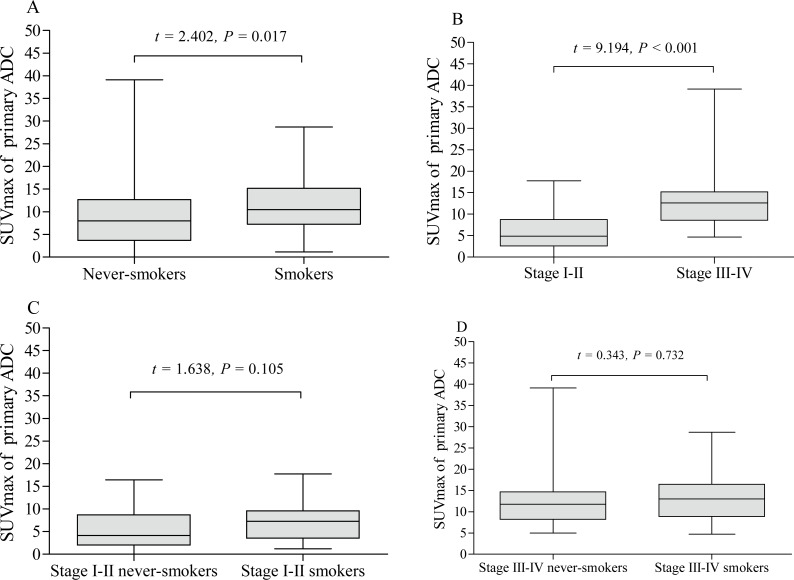
Comparative analysis of pSUVmax in adenocarcinoma with different cigarette smoking history and clinical stage. The pSUVmax of smokers with adenocarcinoma was higher than that of never-smokers (A). Stage III–IV adenocarcinoma had a higher pSUVmax than that in stage I–II adenocarcinoma (B). No significant differences of pSUVmax were observed between stage I–II smokers and never smokers (C), and between stage III–IV smokers and never smokers (D).

**Figure 7:**
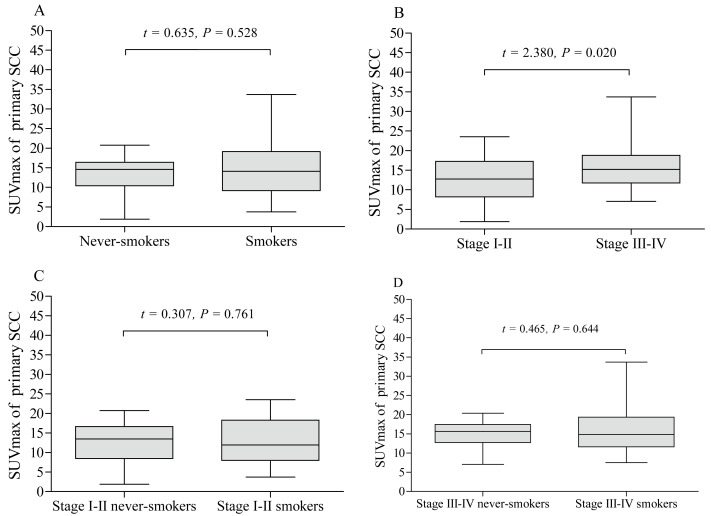
Comparative analysis of pSUVmax in squamous cell carcinoma with different cigarette smoking history and clinical stage. The pSUVmax of smokers with squamous cell carcinoma showed no significant difference compared to never-smokers (A). Stage III–IV squamous cell carcinoma had a higher pSUVmax than that in stage I–II squamous cell carcinoma (B). No significant differences of pSUVmax were observed between stage I–II smokers and never smokers (C), and between stage III–IV smokers and never smokers (D).

The pSUVmax was significantly different among patients with different pathological subtypes of lung cancer (*F* = 11.45, *P* < 0.001, Fig. 5), while only ADC and SCC group showed significant difference in pSUVmax (*t* = 6.667, *P* < 0.001). Smokers with ADC showed a higher pSUVmax when compared to never-smokers with ADC (11.1 ± 5.8 *vs.* 8.8 ± 6.4; *t* = 2.402, *P* = 0.017, Fig. 6A), but not in SCC (14.4 ± 5.9 *vs.* 13.6 ± 4.9; *t* = 0.635, *P* = 0.528, Fig. 7A). The pSUVmax in stage I–II ADC was found lower than that in stage III–IV ADC (Fig. 6B), and similar result was also found in SCC (Fig. 7B). Interestingly, there were no significant differences of pSUVmax between smokers and never-smokers at stage I–II ADC or SCC (Figs. 6C and 7C) and stage III–IV ADC or SCC (Figs. 6D and 7D).

## Discussion

In the present study, we performed a retrospective analysis to investigate the effects of cigarette smoking on ^18^F-FDG metabolic status of lung cancer and found that the pSUVmax of smokers with early lung cancer was significantly higher than that of never smokers, but not in those of advanced stage. With the increase of cigarette smoking dose, the pSUVmax increased significantly. Our results demonstrated a close association between cigarette smoking and metabolic activity of lung cancer and suggest that smoking may be a potential risk factor of higher pSUVmax in early lung cancer on ^18^F-FDG PET/CT.

Since the 1950s, cigarette smoking has been demonstrated to be a risk factor for all forms of lung cancer according to epidemiological evidence (Toh et al., 2006; Doll & Hill, 1950). Various studies showed that smokers with NSCLC had significantly inferior survival outcome when compared with never-smokers (Nordquist et al., 2004; Kawaguchi et al., 2010a; Kawaguchi et al., 2010b; Takamori et al., 2021). The gender, histopathology and molecular genetics of patients with lung cancer between smokers and never-smokers were proved to be variable (Yano et al., 2008; Toh et al., 2006; Kawaguchi et al., 2010a; Kawaguchi et al., 2010b). A high proportion of female and adenocarcinoma generally observed in lung cancer patients with no cigarette smoking history (Toh et al., 2006; Kawaguchi et al., 2010a; Kawaguchi et al., 2010b). In this regard, our results were consistent with these previous reports. Moreover, several studies showed that there were significant differences in molecular genetics between smokers and non-smokers with lung cancer (Shigematsu & Gazdar, 2006; Pham et al., 2006; Jida et al., 2009; Kosaka et al., 2004). EGFR mutations were more common in people who never smoked than in smokers, with an incidence of 45% mutations in never-smokers and 7% in tobacco associated lung cancer (Shigematsu & Gazdar, 2006). Lung cancer in non-smokers and tobacco-associated lung cancer seems to be two distinct entities owing to the strikingly different EGFR mutation status and clinicopathological features (Subramanian & Govindan, 2008).

In addition, a non-invasive method, ^18^F-FDG PET-CT, has been evaluated comprehensively to predict EGFR mutation status in patients with NSCLC (Lv et al., 2018; Liu et al., 2020; Mu et al., 2020). Generally, low pSUVmax, nSUVmax and mSUVmax were significantly correlated with EGFR mutation status in patients with NSCLC (Lv et al., 2018). Lee et al. reported that low mSUVmax favors the presence of EGFR mutations in stage IV lung adenocarcinoma (Lee et al., 2015). Besides, Mu et al. established a non-invasive model based on ^18^F-FDG-PET/CT deep learning, which achieved high accuracy in predicting EGFR mutation status (Mu et al., 2020). Therefore, a close correlation between the metabolic activity of lung cancer on ^18^F-FDG PET/CT and EGFR mutations was formed naturally. However, there is a lack of reports to illustrate the relationship between cigarette smoking history and ^18^F-FDG metabolic status on PET/CT in patients with lung cancer. To our knowledge, only Na et al. reported a similar result that pSUVmax in never-smokers was lower than that in smokers, but they just presented it as an interesting phenomenon and did not further evaluate the correlation between them (Na et al., 2008).

In this study, we evaluated the effects of cigarette smoking on metabolic status of lung cancer on baseline ^18^F-FDG PET/CT. We found that pSUVmax in smokers was significantly higher than that in never-smokers. Tobacco smoking is the major risk of lung cancer-related mortality globally, resulting in poorer survival outcomes for lung cancer smokers than never-smokers (Nordquist et al., 2004). In addition, high pSUVmax measured on ^18^F-FDG PET/CT demonstrated to be a poor prognostic factor of survival in patients with NSCLC (Paesmans et al., 2010; Na et al., 2014). Patients with advanced lung cancer had a worse outcome than those in the early stage (Nicholson et al., 2016). At this point, our results showed that patients with advanced lung cancer presented higher pSUVmax than those at early stage, which may indirectly correlated with previous results. Interestingly, there was significant difference of pSUVmax between smokers and never-smokers in early lung cancer but not in advanced stage. Although approximately 60% of patients with lung cancer are diagnosed at advanced stage, the survival outcomes are significantly better among never smokers than those of smokers, including the early stage patients (Nemesure, Albano & Nemesure, 2021). Accordingly, the pSUVmax of smokers was significantly higher than that of never smokers, which reflected that cigarette smoking and/or higher pSUVmax were risk factors for poor survival of patients with lung cancer. In addition, smokers had a higher proportion of advanced lung cancer compared to never smokers, which may be one reason why smokers with lung cancer have a lower survival rate than that of never smokers (Kawaguchi et al., 2010a; Kawaguchi et al., 2010b). However, there was no significant difference of pSUVmax between smokers and never-smokers in stage I–II ADC or SCC and stage III–IV ADC or SCC. This may be due to the small sample size in the subgroup analysis in our study. Overall, cigarette smoking may be a potential risk factor of higher pSUVmax for patients with early stage lung cancer.

Cumulative smoking dose, described in terms of pack-years, was assessed for its association with pSUVmax in our study. With the increase of the number of cigarette pack-years, the uptake of ^18^F-FDG in primary lung cancer increased remarkably (*r* = 0.217). Jida et al. (2009) reported that the OS and PFS rates were significantly higher in smokers who smoked less than 13 pack-years than those smoked equal or more than 13 pack-years. Accordingly, the risk of survival outcome might be increased with the increase of smoking dose. But in our results, the pSUVmax in smokers showed no significant difference between smoking dose less than 40 pack-years and those equal or more than 40 pack-years. Moreover, the effect of cigarette smoking revealed no significant difference on nSUVmax and mSUVmax between smokers and never-smokers, even with the increase of cigarette smoking dose. The results may indicate that cigarette smoking has no effect on the metabolic activities of metastatic lymph nodes and distant metastasis.

Besides the nature of retrospective analysis, our study has some other limitations. First, compared to previous studies (Kawaguchi et al., 2010a; Kawaguchi et al., 2010b), the number of patients with cigarette smoking history enrolled in our analysis is relatively small, especially in the subgroup analysis. Second, we just analyzed the pSUVmax, nSUVmax and mSUVmax to reflect metabolic status for lung cancer patients, which could not reflect tumor burden for further analysis. Third, due to the lack data on EGFR mutation status, it is impossible to evaluate the relationship between EGFR mutation status, cigarette smoking history and ^18^F-FDG metabolic activity. Last but not least that the survival of our patients was not evaluated, although many studies have shown that the prognosis of lung cancer patients who smoke is lower than that of never-smoking patients, and the prognosis of patients with high pSUVmax is lower than that of patients with low pSUVmax. Accordingly, further large-sample studies are needed to validate our findings and profoundly illustrate the effects of cigarette smoking on metabolic activity on ^18^F-FDG PET/CT for patients with lung cancer.

## Conclusions

In summary, our preliminary findings showed cigarette smoking significantly increased the metabolic activity of primary early lung cancer on baseline ^18^F-FDG PET/CT, suggesting that smoking may be a potential risk factor of higher pSUVmax on PET/CT in patients with early lung cancer. Moreover, cigarette smoking may have no effect on glucose metabolism in metastatic lymph nodes or distant metastasis. Cigarette smoking dose was highly correlated with the level of pSUVmax, which may contribute to understanding of the difference in metabolic activity of lung cancer on ^18^F-FDG PET/CT between smokers and never-smokers.

## Supplemental Information

10.7717/peerj.13352/supp-1 Supplemental Information 1 Raw data

## Additional Information and Declarations

### Data Availability

The following information was supplied regarding data availability: The raw measurements are available in the Supplementary File.
